# Cell-Penetrant, Nanomolar *O*-GlcNAcase Inhibitors Selective against Lysosomal Hexosaminidases

**DOI:** 10.1016/j.chembiol.2010.09.014

**Published:** 2010-11-24

**Authors:** Helge C. Dorfmueller, Vladimir S. Borodkin, Marianne Schimpl, Xiaowei Zheng, Robert Kime, Kevin D. Read, Daan M.F. van Aalten

**Affiliations:** 1 Division of Molecular Microbiology, College of Life Sciences, University of Dundee, Dundee DD1 5EH, Scotland; 2 Division of Biological Chemistry and Drug Discovery, College of Life Sciences, University of Dundee, Dundee DD1 5EH, Scotland

## Abstract

Posttranslational modification of metazoan nucleocytoplasmic proteins with N-acetylglucosamine (*O*-GlcNAc) is essential, dynamic, and inducible and can compete with protein phosphorylation in signal transduction. Inhibitors of *O*-GlcNAcase, the enzyme removing *O*-GlcNAc, are useful tools for studying the role of *O*-GlcNAc in a range of cellular processes. We report the discovery of nanomolar OGA inhibitors that are up to 900,000-fold selective over the related lysosomal hexosaminidases. When applied at nanomolar concentrations on live cells, these cell-penetrant molecules shift the *O*-GlcNAc equilibrium toward hyper-*O*-GlcNAcylation with EC_50_ values down to 3 nM and are thus invaluable tools for the study of *O*-GlcNAc cell biology.

## Highlights

► Structure-guided design of human *O*-GlcNAcase inhibitors, GlcNAcstatins ► The GlcNAcstatins are competitive, nanomolar inhibitors ► The molecular basis of the exquisite selectivity revealed by crystallography ► First direct evidence of *O*-GlcNAcase inhibitors penetrating cells

## Introduction

The *O*-GlcNAc modification was discovered more than two decades ago by Torres and Hart (1984). This modification consists of a single GlcNAc sugar that is dynamically and reversibly transferred onto/hydrolyzed from serine/threonine residues on proteins in the nucleocytoplasm (Hart et al., 2007; Hanover et al., 2010). Similar to protein phosphorylation, the *O*-GlcNAc modification has been shown to be involved in signaling processes and to occupy serine/threonine residues identical, or adjacent, to known protein phosphorylation sites (Roquemore et al., 1996; Chou et al., 1992; Zachara and Hart, 2006; Hart et al., 2007). *O*-GlcNAc transfer is performed by a single enzyme, the *O*-GlcNAc transferase (OGT) (Kreppel et al., 1997; Lubas et al., 1997). The *O*-GlcNAc modification is removed by a single enzyme termed *O*-GlcNAcase (OGA) (Gao et al., 2001).

A range of approaches has been used to gain insight into the function of *O*-GlcNAc and its possible regulation of signaling pathways, including genetic manipulation (Bowe et al., 2006; Ngoh et al., 2009) and blocking *O*-GlcNAc hydrolysis with small molecules that inhibit hOGA. A number of hOGA inhibitors have been described and used to demonstrate the involvement of *O*-GlcNAc in the insulin response (Akimoto et al., 2007), regulation of Tau phosphorylation (Yuzwa et al., 2008), Akt activation (Vosseller et al., 2002), and, recently, in regulation of cellular volume (Nagy et al., 2009). However, many of these inhibitors possess limited selectivity over the structurally related lysosomal hexosaminidases A/B (HexA/B) and/or are only active on cells in the high micromolar range, increasing the risk of off-target effects, as suggested recently by Macauley et al. (2008), Yuzwa et al. (2008), and Dorfmueller et al. (2006, 2009).

Here we have attempted to target a conserved hOGA active site cysteine with suicide inhibitors, based on the GlcNAcstatin scaffold. These novel compounds are up to 900000-fold selective over HexA/B, penetrating the OGA active site as revealed by X-ray crystallography. The compounds penetrate live cells, inducing hyper-*O*-GlcNAcylation when used at low nanomolar concentrations. The new GlcNAcstatins will be useful tools for studying the role of *O*-GlcNAc in cellular signaling pathways.

## Results and Discussion

### Design of Novel GlcNAcstatin-Based Suicide OGA Inhibitors

**Figure 1:**
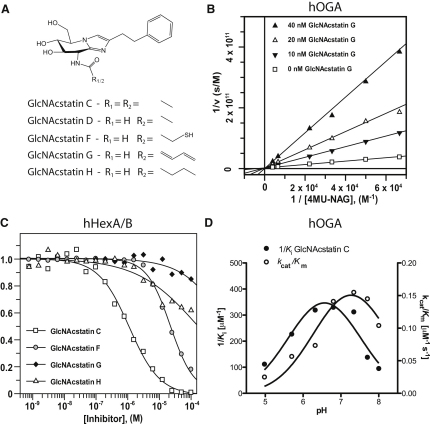
GlcNAcstatins and Their Inhibitory Activities (A) Chemical structures of GlcNAcstatins C, D, and F–H. (B) Lineweaver-Burk analysis of hOGA steady-state kinetics measured in the presence of 0–40 nM GlcNAcstatin G at pH 7.3. Data were fitted using the standard equation for competitive inhibition in the GraFit program (Leatherbarrow, 2001), yielding a K_i_ of 4.1 nM (Table 1). (C) Dose-response curve of hHexA/B inhibition GlcNAcstatins C and F–H. Data were fitted using the standard IC_50_ equation in the GraFit program (Leatherbarrow, 2001). (D) Characterization of pH optimum of hOGA catalytic activity (open circles) and GlcNAcstatin C inhibition (black dots). The catalytic activity was measured using a McIlvaine buffer system over a 4.9–8.1 pH range. Data for 1/K_i_ and k_cat_/K_m_ were plotted versus the pH and fitted by nonlinear regression to the bell-shaped double pK_a_ equation in the program GraphPad Prism. The pH optimum for hOGA hydrolytic activity is pH 7.3 (right *y*-axis), and the pH optimum GlcNAcstatin C inhibition is at pH 6.6 (left *y*-axis).

**Figure 2:**
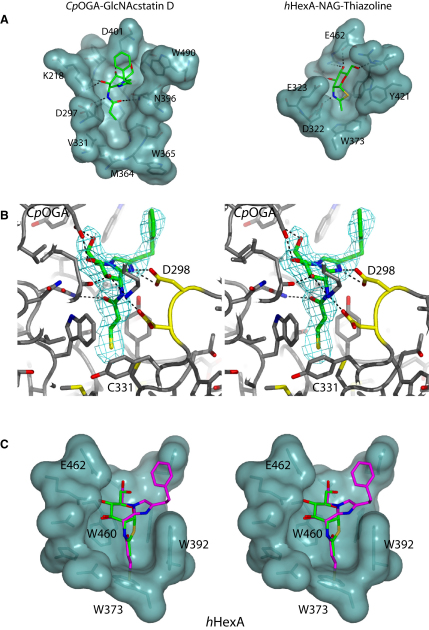
Binding of GlcNAcstatins to *Cp*OGA (A) Comparison of the active-site architecture of OGA enzymes and hexosaminidases. The active site of *Cp*OGA in complex with GlcNAcstatin D (PDB entry 2WB5) (Dorfmueller et al. [2009]) is shown in a semitransparent surface representation. GlcNAcstatin D is shown in sticks with green carbon atoms. hHexA in complex with NAG-thiazoline (PDB entry 2GK1) (Lemieux et al. [2006]) is shown with NAG-thiazoline in sticks with green carbon atoms. The residues blocking the active site from this side view (Tyr335 in *Cp*OGA and Trp392 in hHexA) have been removed in these images for clarity. Hydrogen bonds between the ligands and active site residues are indicated by black dashed lines. (B) Stereo figure of the crystal structure of GlcNAcstatin F (sticks with green carbon atoms) in complex with V331C-*Cp*OGA. Hydrogen bonds are indicated by black dashed lines. An unbiased |F_o_ |− |F_c_ |, φ_calc_ electron density map calculated without the model having seen the inhibitor in refinement is shown at 2.75 σ. (C) Stereo figure of a superimposition of GlcNAcstatin F onto the hHexA-thiazoline complex. Semitransparent surface representation of hHexA in complex with NAG-thiazoline (green carbon atoms) (PDB entry: 2GK1) (Lemieux et al. [2006]). GlcNAcstatin F (magenta carbon atoms) is superimposed onto NAG-thiazoline.

GlcNAcstatins (Figure 1A), a novel family of potent human OGA inhibitors, have recently been reported to possess 150-fold selectivity over hHexA/B (Dorfmueller et al., 2006, 2009). Based on the structural data of GlcNAcstatins in complex with a bacterial OGA from *C*. *perfringens* (*Cp*OGA) (Figure 2A), we assumed that increased selectivity for hOGA over the HexA/B could be achieved by extending the size of the *N*-acyl derivative. However, similar elaboration of the NAG-thiazoline/PUGNAc scaffolds reduced inhibition from nanomolar to micromolar range (Macauley et al., 2005; Stubbs et al., 2006). A structural comparison of the active site pockets from *Cp*OGA (Dorfmueller et al., 2009) and hHexA (Lemieux et al., 2006) and hHexB (Mark et al., 2003) reveals obvious differences in the *N*-acetyl binding pocket (Figure 2A). The β-hexosaminidases have a narrower (difference of approximately 1.4 Å) and shallower (difference of approximately 5.0 Å) pocket than the OGA enzymes (Figure 2A). Interestingly, a cysteine residue is located at the bottom of the hOGA active site (Cys215) (Figure 2B). This cysteine is conserved in metazoan OGAs, and hOGA is potently inhibited by a thiol-reactive compound (Dong and Hart, 1994).

In an attempt to generate a potent, selective hOGA “suicide” inhibitor, the *N*-acyl group of GlcNAcstatin D was extended and modified to contain thiol-reactive groups that could irreversibly react with the cysteine located in a pocket at the bottom of the active site. GlcNAcstatin F carries a 3-mercaptopropanamide side chain (Figure 1A) and GlcNAcstatin G a penta-2,4-dienamide derivative, both potentially able to react with the hOGA Cys215. GlcNAcstatin H, a saturated derivative of GlcNAcstatin G, was synthesized as a control (Figure 1A). The synthesis will be reported elsewhere.

### GlcNAcstatins F–H Show Increased hOGA Selectivity while Retaining Potency

The new GlcNAcstatin derivatives were evaluated in kinetic studies for their ability to inhibit recombinant hOGA. The pH optimum of hOGA is 7.3 (Figure 1D), whereas the first GlcNAcstatin inhibitor reported (GlcNAcstatin C) inhibits with maximum potency at pH 6.6 (K_i_ = 2.9 nM) (Figure 1D). At pH 7.3, GlcNAcstatins F–H show time-independent inhibition in the 2.6–11.2 nM range (Table 1 and Figures 1A and 1B). To assess selectivity, inhibition of hHexA/B was also investigated (Figure 1C). The extension of the *N*-propionyl side chain of GlcNAcstatin D with an additional thiol group (GlcNAcstatin F) increases selectivity for hOGA to 1000-fold (Figure 1C and Table 1), showing that the elongated *N*-acyl substitution abolishes the binding of the compound to hHexA/B (Table 1). Strikingly, the more extended GlcNAcstatin G inhibits hHexA/B with an approximate IC_50_ of only 7 mM (Figure 1C and Table 1), thus resulting in a >900,000-fold selectivity for GlcNAcstatin G toward hOGA, representing the most selective hOGA inhibitor reported to date.

### The 3-Mercaptopropanamide Side Chain of GlcNAcstatin F Efficiently Occupies the Selectivity Pocket

The molecular basis of the increased selectivity of the new GlcNAcstatin derivatives was investigated by X-ray crystallography using the *Cp*OGA V331C mutant that perfectly mimics the hOGA active site (Dorfmueller et al., 2009) (Figure 2B). The *Cp*OGA-V331C GlcNAcstatin F complex (2.5 Å resolution) reveals that the inhibitor-binding mode is similar to the previously reported GlcNAcstatin C/D complexes (Dorfmueller et al., 2006, 2009) (Figure 2B). The 3-mercaptopropanamide moiety points straight into the pocket of the active site, whereas all hydrogen bonds seen in the GlcNAcstatin C/D complexes are conserved. Interestingly, the sulfhydryl group of GlcNAcstatin F approaches the active site cysteine (Cys215 in hOGA, equivalent to Cys331 in the V331C-*Cp*OGA mutant) to within 3.5 Å (Figure 2B), although the side chain thiol points away from the inhibitor, and no disulfide bond is formed, in agreement with the absence of time-dependent inhibition. A structural superposition of the GlcNAcstatin F-V331C-*Cp*OGA complex onto the structure of hHexA (Figure 2C) reveals that the shallower *N*-acetyl binding pocket in the HexA structure would produce steric clashes with the 3-mercaptopropanamide moiety, also explaining the increased selectivity for hOGA of >900,000-fold for the further elongated penta-2,4-dienamide substituent (GlcNAcstatin G).

### GlcNAcstatin G Penetrates HEK293 Cells

No direct evidence has so far been provided for any known hOGA inhibitor penetrating the cell and inducing cellular hyper-*O*-GlcNAcylation by direct inhibition of intracellular hOGA. We have used a mass-spectrometric approach to measure intracellular GlcNAcstatin G concentrations in HEK293 cells. HEK293 cells were treated in triplicate with two inhibitor concentrations (1 and 10 μM) for 1.5 and 6 hr. An equal number of cultured cells were lysed, and the intracellular volume was investigated by UPLC-MS/MS. On the basis of the average cell size (13.1–15.7 μm) and the number of cultured cells (1.4 × 10^6^ to 2.2 × 10^6^), the maximal cellular volume and, thus, the minimal dilution factor of the intracellular volume upon the addition of lysis buffer were calculated (280-fold). Treatment of HEK293 cells with 10 μM GlcNAcstatin G resulted in a minimal inhibitor concentration of 270 nM after 1.5 hr and 380 nM after 6 hr. Application of a 1 μM extracellular concentration resulted in a 10-fold lower intracellular concentration of at least 30 and 40 nM, after 1.5 and 6 hr treatment, respectively. To our knowledge, these data provide the first direct evidence that GlcNAcstatins are cell-penetrant compounds.

### GlcNAcstatins Effectively Induce Cellular Hyper-*O*-GlcNAcylation at Low Nanomolar Concentrations

**Figure 3:**
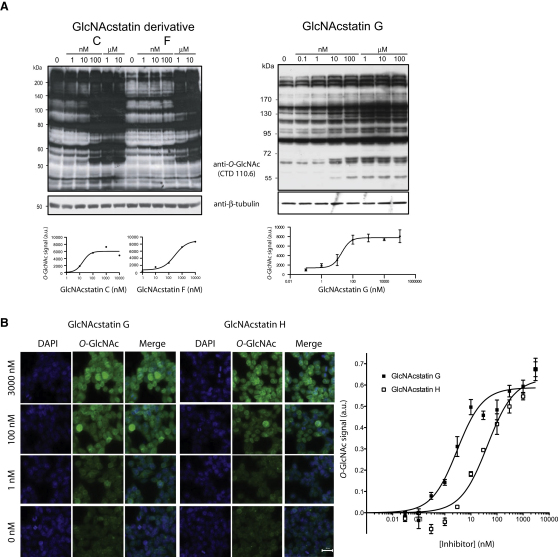
hOGA Inhibitors GlcNAcstatins G and H Elevate *O*-GlcNAc Levels in HEK293 Cells (A) HEK293 cells were incubated with GlcNAcstatins C, F, and G for 6 hr at a range of doses. Cellular *O*-GlcNAcylation levels were qualitatively analyzed using an *O*-GlcNAc antibody. Densitometric quantitation of single western blots (GlcNAcstatins C and F) and of three *O*-GlcNAc western blots (GlcNAcstatin G) reveals EC_50_ values of 20 nM for GlcNAcstatin C, 290 nM for GlcNAcstatin F, and 20 nM for GlcNAcstatin G. (B) Immunostaining using an *O*-GlcNAc antibody shows an elevation of total *O*-GlcNAc modification (green) of HEK293 cells. Increased concentrations of GlcNAcstatins G and H induce hyper-*O*-GlcNAcylation. Dose-response curves of GlcNAcstatin treatments demonstrate the efficiency of the inhibition. Images were analyzed with the IN cell analyzer, and *O*-GlcNAc signal in each frame was normalized over the DAPI signal (blue). The scale bar represents 20 μm. See also Figure S1.

The novel GlcNAcstatin derivatives were investigated for their activity on endogenous hOGA in a cell-based assay with HEK293 cells. Cells were exposed for 6 hr to a range of inhibitor concentrations and investigated for an increase of cellular *O*-GlcNAcylation levels by western blotting using an anti-*O*-GlcNAc antibody (CTD110.6), followed by the fitting of densitometric data to a standard dose-response curve (Figure 3A), yielding EC_50_s of 20 nM for both GlcNAcstatins C and G, and an EC_50_ of 290 nM for GlcNAcstatin F. Treatment with concentrations as low as 100 nM of GlcNAcstatin G in the culture media appears to already induce maximum levels of *O*-GlcNAcylation (Figure 3A). Furthermore, we determined the EC_50_ of GlcNAcstatins G and H by *O*-GlcNAc microscopy assay (Figure 3B; see Figure S1 available online). Elevated total *O*-GlcNAc levels in HEK293 cells were quantified and normalized over a reference DAPI signal. Dose-response curves of these analyses yielded EC_50_ values of 3 and 42 nM for GlcNAcstatins G and H, respectively, in good agreement with the immunoblotting-derived data (Figures 3A and 3B).

In conclusion these new compounds and, in particular, GlcNAcstatin G provide potent and selective chemical biological dissection tools as an attractive alternative to genetic approaches for modulating intracellular *O*-GlcNAc levels in cells to study the role of *O*-GlcNAc in signal transduction.

## Significance

**Selective inhibition of human *O*-GlcNAcase is a useful strategy to study the role of the *O*-GlcNAc modification in living cells. Previously reported compounds showed undesirable inhibition of the human lysosomal hexosaminidases, and there has been no direct evidence of the cell penetration of these compounds. This work reports derivatives of the GlcNAcstatin scaffold, showing potent inhibition of human *O*-GlcNAcase with up to 900,000-fold weaker inhibition of the lysosomal hexosaminidases. These are the most selective human *O*-GlcNAcase inhibitors known to date and penetrate live cells to induce cellular hyper-*O*-GlcNAcylation levels with EC_50_s in the low nanomolar range.**

## Experimental Procedures

### Determination of the *Cp*OGA-GlcNAcstatin F Complex Crystal Structure

Cloning, expression, purification, and crystallization of hOGA and/or *Cp*OGA have been described previously (Dorfmueller et al., 2009). Diffraction data for a *Cp*OGA-V331C GlcNAcstatin F complex were collected on BM-14 (ESRF, Grenoble) to 2.4 Å (Table 2). Refinement to 2.5 Å resolution was initiated from the protein model in the *Cp*OGA-GlcNAcstatin C complex (PDB entry 2J62) (Dorfmueller et al. [2006]), and completed by iterative model building using COOT (Emsley and Cowtan, 2004) and refinement with REFMAC (Murshudov et al., 1997) (Final R, R_free_ at 2.5 Å resolution: 0.197, 0.236).

### Inhibitor Potency and Mode of Action

Steady-state kinetics (measurements were performed in triplicate) and inhibition constants (K_i_) for GlcNAcstatin derivatives were determined using the fluorogenic assay as described previously (Dorfmueller et al., 2009).

The mode of inhibition was visually verified by a Lineweaver-Burk plot (Figure 1B) and the K_i_ determined by fitting all fluorescence intensity data to the standard equation for competitive inhibition in GraFit (Leatherbarrow, 2001) (Table 1). IC_50_ measurements with hHexA/B (Sigma A6152) activities against GlcNAcstatins F, G, and H were performed using the fluorogenic 4MU-NAG substrate and standard reaction mixtures as described previously (Dorfmueller et al., 2009) (Figure 1C).

### Cell-Based Assays and Western Blots

For the *O*-GlcNAc western blots, HEK293 cells were cultured and treated with GlcNAcstatin derivatives as described previously (Dorfmueller et al., 2009). Cell lysates were separated by SDS PAGE, and *O*-GlcNAcylation was detected by western blotting with the anti-*O*-GlcNAc CTD110.6 antibody and quantified (Dorfmueller et al., 2009). To calculate the EC_50_ values of GlcNAcstatins C, F, and G, the data, background corrected with the untreated cells, were plotted against the inhibitor concentrations using the program GraphPad Prism (http://www.graphpad.com).

For the microscopy studies, HEK293 cells were seeded in clear flat-bottom 96 well assay plates (Corning 3340) and grown for 12 hr at 37°C under 5% CO_2_. Cells were treated with concentrations of GlcNAcstatins G and H (1% final DMSO concentration) for 6 hr. Cells were washed twice with 50 μl PBS and fixed in 3.7% formaldehyde for 10 min at 37°C. After washing with TBST (0.1% Triton X-100) and permeabilized for further 10 min, the cells were blocked with 2% BSA and 0.1% normal donkey serum in TBST for 1 hr at RT, followed by washing twice with TBST. Cells were immunostained with the RL2 *O*-GlcNAc antibody (1/500) for 12 hr at 4°C and fluorescent secondary (1/500) for 1 hr at RT. Cells were further washed twice with TBS before incubation with DAPI (5 μg/ml) and CellMask (1/25000). Cells were washed twice with TBS and stored in 50 μl PBS. Images were taken and analyzed with the IN Cell Analyzer 1000 system (GE Healthcare).

### Determination of Intracellular GlcNAcstatin G Concentration

HEK293 cells were treated as described above with two concentrations (1 and 10 μM) of GlcNAcstatin G for 1.5 and 6 hr. Cells were washed twice in ice-cold PBS and harvested in lysis buffer. The soluble fraction was separated from cell debris by centrifugation and direct analysis performed by UPLC-MS/MS on a Quattro Premier XE mass spectrometer using positive electrospray ionization (Waters, UK) in multiple reaction-monitoring mode. A calibration curve was constructed in lysis buffer to cover approximately 3 orders of magnitude for GlcNAcstatin G (i.e., 0.25–500 nM). To calculate the minimal intracellular concentration of GlcNAcstatin G, the number and average size (diameter in μm) of cultured HEK293 cells that were used in this study were analyzed using a Cellometer Auto T4 from Nexcelom Bioscience. Subsequently, the maximal intracellular volume was approximated as V = (4/3) × π × r^3^, assuming a spherical cell and ignoring effects of intracellular compartments and proteins. To calculate the total maximal volume of the intracellular volume in a 2 ml culture, the total number of HEK293 cells was calculated and multiplied by the average single-cell volume. This volume was used to determine the minimal dilution factor of the intracellular volume upon cell lysis. To determine the cellular uptake and, thus, the minimal concentration of freely available GlcNAcstatin G, the concentration of the compound determined by UPLC-MS/MS was corrected using the dilution factor.

## Figures and Tables

**Table 1:** Inhibition Data and Selectivity of GlcNAcstatins C and F–H, PUGNAc, and Thiamet-G against Lysosomal hHexA/HexB, Human OGA and *Cp*OGA-WT and V331C-*Cp*OGA Mutant

	K_i_ (μM)	K_i_ (nM)	Selectivity	K_i_ (nM)	K_i_ (nM)
	hHex A/B^a^	hOGA^b^	(hHexA/B/hOGA)	*Cp*OGA-wild-type	V331C-*Cp*OGA
GlcNAcstatin C	0.6 ± 0.1	3.2 ± 0.9	190	0.0046 ± 0.0002^c^	0.098 ± 0.006^c^
GlcNAcstatin F	11.0 ± 0.6^d^	11.2 ± 1.4	1,000	0.0032 ± 0.0002	0.005 ± 0.001
GlcNAcstatin G	>3,700^d^	4.1 ± 0.7	>900,000	0.0078 ± 0.0007	0.019 ± 0.002
GlcNAcstatin H	100 ± 30^d^	2.6 ± 0.3	35,000	nd	nd
PUGNAc	0.036^e^	50^e^	ns	5.4 ± 0.4	nd
Thiamet-G	750^f^	21^f^	35,000	nd	nd

nd, not determined; ns, no selectivity for hOGA.

a The Cheng-Prusoff equation (K_i_ = IC_50_/(1 + [S]/K_m_)) was used to convert the IC_50_ values to an absolute inhibition constant (K_i_).

b At physiological pH 7.3.

c From Dorfmueller et al. (2009).

d These values are approximate IC_50_s, obtained after including an artificial 100% inhibition point at an inhibitor concentration of 1000 M.

e From Macauley et al. (2005).

f From Yuzwa et al. (2008).

**Table 2:** Details of Data Collection and Structure Refinement for GlcNAcstatin F Bound to V331C-*Cp*OGA

	V331C-*Cp*OGA-GlcNAcstatin F
Unit cell (Å)	a = 130.2
	b = 145.0
	c = 153.1
Resolution range (Å)	20.0–2.4 (2.5–2.4)
Number of observed reflections	214,504
Number of unique reflections	53,607 (4,916)
Redundancy	4.0 (3.6)
I/σI	14.7 (2.3)
Completeness (%)	95.9 (88.6)
R_merge_	0.129 (0.606)
Number of protein residues	1,170
Number of water molecules	454
R, R_free_	0.197, 0.235
RMSD from ideal geometry	
—Bonds (Å)	0.01
—Angles (°)	1.3
B-factor RMSD (Å^2^)	
— (backbone bonds)	31.6
<B > (Å^2^)	
—Protein	31.6
—Inhibitor	32.1
—Solvent	32.9

Values in parentheses are for the highest resolution shell. All measured data were included in structure refinement. The space group was I2_1_2_1_2_1_.

## Accession Numbers

Coordinates and structure factors are deposited in the RCSB protein data bank (PDB entry 2XPK).

## Supplemental Information

Document S1. Supplemental Experimental Procedures, Six Figures, and One Table
