# Supplementary material for: Cell-Penetrant, Nanomolar O-GlcNAcase Inhibitors Selective against Lysosomal Hexosaminidases
Source: Chem Biol. 2010 Nov 24;17(11):1250–5. doi: 10.1016/j.chembiol.2010.09.014 (PMC3032886; doi:10.1016/j.chembiol.2010.09.014)

**Chemistry & Biology** ***17***

# Supplemental Information

# Cell-Penetrant, Nanomolar *O*-GlcNAcase Inhibitors Selective against Lysosomal Hexosaminidases

**Helge C. Dorfmueller, Vladimir S. Borodkin, Marianne Schimpl, Xiaowei Zheng, Robert Kime, Kevin D. Read, and Daan M.F. van Aalten**

# Supplementary information

# Supplementare Figure S1

Thumbnail images of immunostained HEK293 cells treated with series concentration of GlcNAc-statin G and H as indicated (0.03 nM to 3 $\mu$M). Immunostaining using an *O*-GlcNAc antibody (RL2) shows an elevation of total *O*-GlcNAc modification (Top) which correlates with the increased concentrations of the inhibitors. The data were collected from experiments carried out in quadruplicate and normalized with DAPI stain (Bottom). Nine frames were shot from each well. The $EC_{50}$ values of GlcNAcstatin G and H were determined and zoom in images at select concentrations are shown in Fig. 3B.

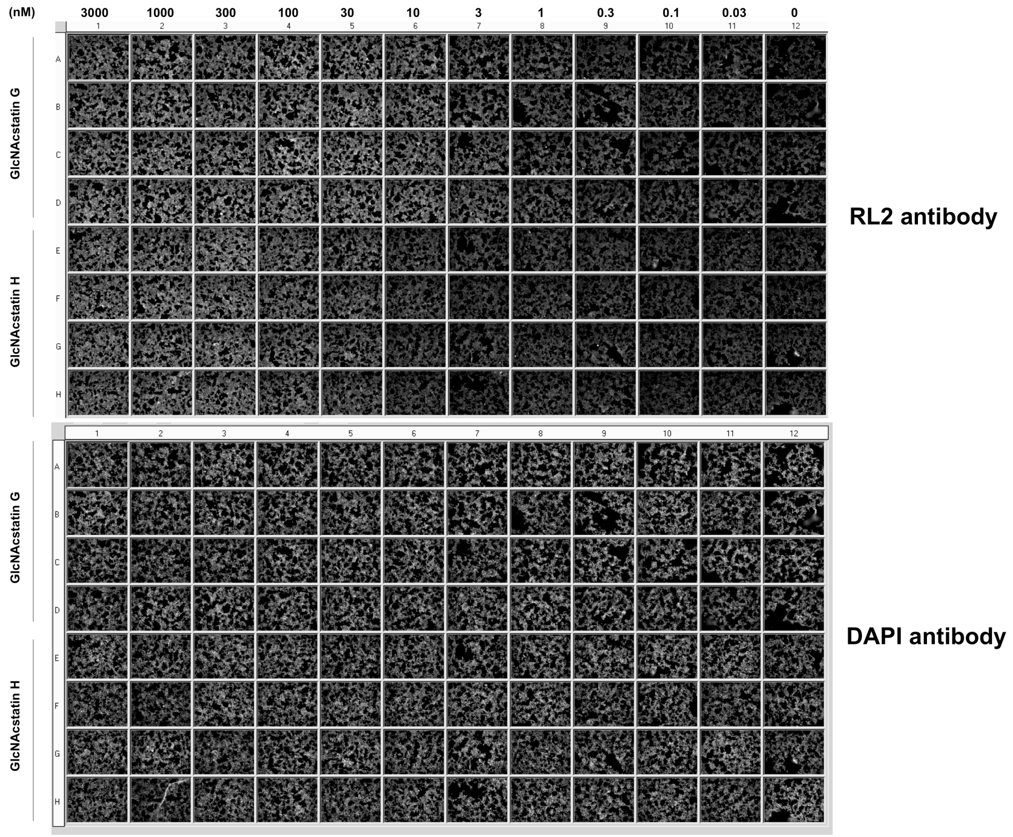

Supplement: Document S1. Supplemental Experimental Procedures, Six Figures, and One Table [file mmc1.pdf]
